# Superior Vena Cava Stenting Complicated by Perforation and Cardiac Tamponade

**DOI:** 10.7759/cureus.78564

**Published:** 2025-02-05

**Authors:** Arusa Macnojia, Jacy Gressen, Claudia Wei, Kevin Tzan, Amy Lee, Jamal Hasoon, Giustino Varrassi, Anvinh Nguyen

**Affiliations:** 1 Anesthesiology, Baylor College of Medicine, Houston, USA; 2 Anesthesia, Critical Care and Pain Medicine, UTHealth, McGovern Medical School, Houston, USA; 3 Pain Medicine, Fondazione Paolo Procacci, Rome, ITA

**Keywords:** cardiac anesthesia, cardiac tamponade, complications, endovascular stenting, superior vena cava syndrome

## Abstract

Superior vena cava (SVC) syndrome is a clinical condition characterized by impaired venous return from the upper body due to intrinsic or extrinsic obstruction of the SVC. Endovascular stenting has become an effective intervention for symptomatic relief. However, the procedure carries a rare risk of life-threatening complications, including SVC perforation and cardiac tamponade. This study describes a 60-year-old male with SVC syndrome secondary to squamous cell carcinoma of the lung who developed SVC perforation and cardiac tamponade during endovascular stenting. Despite initial hemodynamic compromise and cardiac arrest, prompt intervention with pericardiocentesis, aggressive resuscitation, and deployment of a covered stent resulted in a successful outcome within 4 minutes.

This report highlights the critical role of anesthetic management in SVC syndrome cases, emphasizing the importance of airway precautions, hemodynamic stability, and access to inferior venous return for large-volume resuscitation. This case underscores the need for vigilance in recognizing and managing SVC stenting complications. Furthermore, it advocates for a multidisciplinary approach involving anesthesiology, interventional radiology, and cardiothoracic surgery to optimize outcomes.

## Introduction

Superior vena cava (SVC) syndrome constitutes a myriad of signs and symptoms that result from intrinsic or extrinsic obstruction of blood flow through the SVC and consequent increase in venous pressure in the upper extremities. The SVC is a large venous structure that drains deoxygenated blood from the upper body into the right atrium of the heart. It lacks valves, making it particularly susceptible to venous congestion in cases of obstruction. The most common causes are direct invasion or external compression of the SVC by malignancy [1]. While the primary treatment focuses on addressing the underlying cause, endovascular stent placement has become a standard approach for rapid symptom relief. Complications of SVC stent placement are reported in 3-7% of patients, with a rare risk of SVC rupture and cardiac tamponade [2]. Timely recognition and intervention are crucial to reduce mortality associated with these complications. Here, we present a case of SVC stenosis managed with endovascular stenting that was complicated by SVC perforation, cardiac tamponade, and subsequent cardiac arrest.

## Case presentation

**Figure 1:**
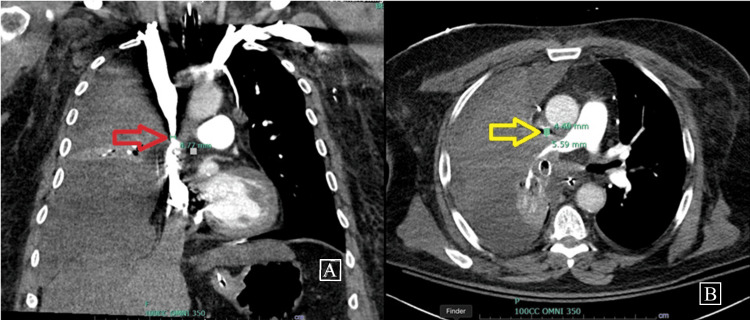
Computed tomography of the chest demonstrating compression of the SVC. The red arrow indicates the site of stenosis in the coronal plane (A), while the yellow arrow highlights the stenosis in the axial plane (B). SVC: superior vena cava

A 60-year-old male with a medical history of type II diabetes mellitus, tobacco use, chronic obstructive pulmonary disease, and persistent atrial flutter presented to the hospital with worsening shortness of breath and fatigue. He also had SVC syndrome secondary to recently diagnosed squamous cell carcinoma of the right lung. Upon arrival at the emergency department, he became unresponsive and cyanotic, necessitating intubation and admission to the ICU. A chest X-ray revealed a large right pleural effusion causing compressive atelectasis of the right lung. Emergent bronchoscopy identified a mucus plug in the right main bronchus, which was suctioned, resulting in improved peak ventilatory pressures. Thoracentesis was subsequently performed, draining 1.5 L of fluid, and a right-sided chest tube was placed. The patient was successfully extubated the following day. Computed tomography of the chest showed circumferential narrowing of the SVC with a minimal diameter of 0.56 x 0.44 cm at the mid-SVC. The computed tomography is shown below (Figures 1A, 1B).

Interventional radiology was consulted and endovascular stenting of the SVC was scheduled. On the day of the procedure, two additional 18-gauge peripheral IVs were placed in the left and right saphenous veins, along with a pre-induction right radial arterial line. Given the patient's impaired venous return from the SVC, IV access was obtained inferiorly to facilitate large-volume resuscitation if needed, ensuring adequate circulatory support in the event of hemodynamic compromise. At the request of the proceduralist, monitored anesthetic care was provided to keep the patient alert and responsive to commands. Oxygen was delivered via a simple face mask. Sedation was maintained with a dexmedetomidine infusion at 0.2 mcg/kg/h, allowing the patient to tolerate venous access through the right femoral vein. Fluoroscopic guidance was used to successfully deploy a 14 x 60 mm Abre stent across the focal stenosis in the central SVC without complications.

**Figure 2:**
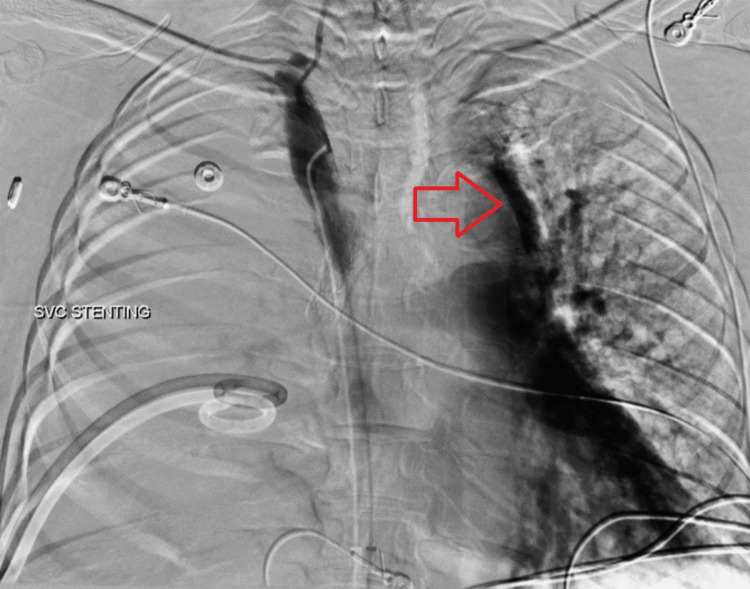
Superior vena cavogram (SVC) post-stent deployment. Superior vena cavogram following stent deployment demonstrating contrast extravasation into the pericardial sac. The red arrow indicates the presence of contrast within the pericardial sac.

However, during balloon angioplasty, the patient experienced panic and dyspnea. A fluoroscopic superior vena cavogram revealed contrast extravasation into the pericardial space, accompanied by a rapid decline in mean arterial pressure and electrical alternans on telemetry. Emergency measures were initiated, including fully opening all IV fluids, calling for blood products, and preparing for massive resuscitation. A balloon catheter was promptly advanced to the site of extravasation and inflated by the interventional radiology team. Cardiothoracic surgery and additional anesthesiology team members were urgently paged, while the interventional radiology team attempted fluoroscopic placement of a pericardial drain. The superior vena cavogram is shown below (Figure 2).

The patient continued to deteriorate rapidly, culminating in a loss of pulses. Emergent intubation and aggressive resuscitation were initiated following advanced cardiac life support (ACLS) protocols. Using ultrasound guidance, a 10-French drain was inserted into the pericardial space. Return of spontaneous circulation (ROSC) was achieved after two rounds of cardiopulmonary resuscitation and the aspiration of approximately 800 cc of blood from the pericardial space. The patient received transfusions of 4 units of packed red blood cells, 2 units of fresh frozen plasma, and 1 unit of platelets.

An intraoperative transesophageal echocardiogram (TEE), performed after successful pericardial drainage and ROSC, revealed minimal residual pericardial effusion and proper placement of the pericardial drain. The balloon catheter in the SVC was deflated, and an 11 x 39 mm covered VBX stent was deployed across the site of the SVC perforation. Digital subtraction angiography confirmed the absence of contrast extravasation.

**Figure 3:**
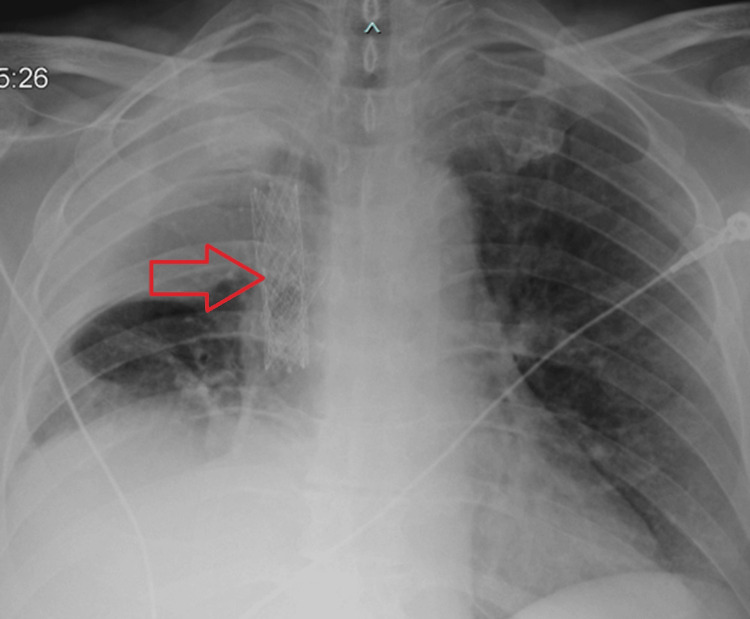
Chest X-ray depicting VBX stent placement. Chest X-ray illustrating VBX stent placement in the superior vena cava. The red arrow indicates the location of the VBX stent.

Post-operatively, the patient remained intubated and sedated before being transferred to the ICU for recovery. He was successfully extubated 48 hours later, and the pericardial drain was removed five days after placement. The patient was discharged from the hospital on post-operative day 14, with no neurological deficits or other complications. A chest X-ray demonstrating the VBX stent is shown below (Figure 3).

## Discussion

The pathology of SVC syndrome is most commonly secondary to external compression or direct invasion of the SVC by malignancy, leading to venous hypertension responsible for the clinical manifestations of the syndrome (e.g., facial, neck, and upper extremity edema, dyspnea, cough). Traditionally, radiation therapy and chemotherapy have been used to manage malignancy-related SVC syndrome. However, endovascular therapy has become the standard practice as it offers more rapid symptom relief. Unlike radiation therapy, endovascular stenting typically does not cause the transient worsening of symptoms associated with radiation therapy and can be performed prior to a biopsy of the causative mass since it does not interfere with histologic sampling. Although there are no randomized controlled trials studying endovascular stenting for the treatment of SVC syndrome, numerous observational studies suggest high rates of successful revascularization (between 80% and 98%) and low procedural complication rates [3]. The cumulative incidence of major complications, such as pericardial tamponade, SVC rupture, stent migration, cardiac injury, and pulmonary edema, is reported to be less than 8%. Pericardial tamponade is exceedingly rare, occurring in only 0.1-1.8% of cases [4].

SVC stent insertion can be performed under moderate sedation or general anesthesia. The procedure involves obtaining central venous access, usually via the femoral vein and less commonly through the internal jugular vein or brachial vein. A large vascular sheath catheter is introduced into the vein, and a navigation wire is inserted. Once the target area of stenosis is reached, venography is performed to determine the appropriate size of the stent to be used. The stent is advanced using the vascular sheath catheter and deployed at the site of the stenosis. Balloon angioplasty may be performed before or after stenting to dilate the area of stenosis and allow remodeling of the stent. At the end of the procedure, a repeat venogram is obtained to confirm the position of the stent. Proper sizing and positioning of the stent are crucial to preventing complications such as vessel injury or stent migration.

A wide range of stents is available for the stenting of the SVC. They can largely be grouped into balloon-expandable vs. self-expanding and covered vs. uncovered. While self-expanding stents reach a predetermined size upon deployment, balloon-expandable stents are expanded through operator-controlled inflation. Balloon-expandable stents may have a higher risk of migration or restenosis because they are more compressible [5]. Covered stents differ from uncovered stents in that they are typically coated with expanded polytetrafluoroethylene (ePTFE). Although covered stents carry a risk of migration and may occlude branches of the SVC or collateral circulation depending on placement, their ePTFE membrane makes them an appropriate choice for controlling vascular perforations [6].

Currently, no randomized studies demonstrate differences in complication rates among the various types of stents used in the treatment of SVC syndrome. However, a retrospective study in patients with malignant SVC syndrome suggests that covered stents have higher cumulative patency rates over 12 months compared to uncovered stents [6].

SVC syndrome presents significant challenges during intraoperative management, particularly concerning airway and hemodynamic stability. Distortion and edema of the airway caused by SVC syndrome can complicate intubation. Oropharyngeal edema may obscure glottic views and increase the risk of airway trauma, which can be severe due to venous engorgement. Positioning patients in a head-up position helps reduce airway edema and facilitates airway securement [7]. Airway obstruction or collapse during anesthesia induction is a serious concern. General anesthesia can reduce lung volumes and relax bronchial smooth muscle, exacerbating existing airway compression. Positive pressure ventilation further decreases transpleural pressure, reducing tracheobronchial diameter. To mitigate these risks, cautious use of sedatives and muscle relaxants along with maintenance of spontaneous ventilation when feasible is recommended [8]. Awake fiberoptic intubation may be considered to preserve spontaneous ventilation and ensure airway patency. This approach allows visualization of airway obstruction and optimal placement of the endotracheal tube distal to the site of narrowing.

Obstruction of the SVC also reduces preload and cardiac output, which can be worsened by anesthetic agents due to vasodilation and further preload reduction. Positive pressure ventilation increases intrathoracic pressure, further impairing venous return and exacerbating hypotension [7]. Management principles, including the mnemonic "fast, full, and tight," focus on preserving preload and cardiac output through generous volume resuscitation and allowing sinus tachycardia to compensate for stroke volume loss. For IV access, lower extremity access is preferred over upper extremity sites, given the interruption of venous return from the head, neck, and upper extremities due to SVC obstruction. Adequate IVC access should also be considered for large-volume resuscitation [9]. It is also very important to preserve the circulation of the spinal cord, which can be done with the appropriate use of epidural corticosteroids [10].

Prompt recognition of SVC injury and cardiac tamponade is critical for optimizing outcomes. Sudden-onset tachycardia and hypotension unresponsive to vasopressors should raise suspicion for hemopericardium. Low-voltage tracings and electrical alternans on an electrocardiogram further support the diagnosis. Common symptoms of tamponade due to SVC rupture include dyspnea, chest pain, and diaphoresis [11].

Successful management of SVC rupture and cardiac tamponade requires restoring cardiac output and addressing the perforation. Rapid pericardiocentesis can be lifesaving but must be performed cautiously, as it removes circulating blood volume without preventing re-accumulation. If available, an occlusion balloon can be used as a temporizing measure while awaiting definitive endovascular therapy [11]. In the present case, covered stenting was employed, and open surgical repair was unnecessary. However, there have been cases where surgical intervention, including open thoracotomy, has been required [12]. Given this information, cardiothoracic surgery should always be notified in case open repair becomes required. Currently, no data compares surgical intervention with conservative management using covered stenting.

## Conclusions

Endovascular stenting has become the standard treatment for SVC syndrome, offering high success rates and rapid symptom relief. However, as demonstrated in this study, serious complications such as SVC perforation and cardiac tamponade, though rare, can be life-threatening. Anesthetic management of patients undergoing SVC stenting requires careful planning and preparation to address potential airway compromise, hemodynamic instability, and challenges in obtaining venous access. Prompt recognition of SVC injury and cardiac tamponade is critical and necessitates a multidisciplinary approach involving interventional radiology, cardiothoracic surgery, and anesthesiology. Early imaging assessment and intervention with pericardiocentesis and definitive management with covered stenting can result in favorable outcomes, as demonstrated in the present case.

Further research is needed to better define optimal management strategies for SVC syndrome and its complications. Prospective studies comparing different stent types and approaches, as well as the role of surgical intervention versus endovascular repair, would provide valuable insights to improve patient outcomes in this challenging clinical scenario.
